# Exercise and the Risk of Dementia in Patients with Newly Diagnosed Atrial Fibrillation: A Nationwide Population-Based Study

**DOI:** 10.3390/jcm10143126

**Published:** 2021-07-15

**Authors:** Jaehyun Lim, So-Ryoung Lee, Eue-Keun Choi, Kyung-Do Han, Jin-Hyung Jung, Hyo-Jeong Ahn, Jun Pil Yun, Soonil Kwon, Seil Oh, Gregory Y. H. Lip

**Affiliations:** 1 Department of Internal Medicine, Seoul National University Hospital, Seoul 03080, Korea; daniellim32@naver.com (J.L.); minerva1368@gmail.com (S.-R.L.); hyojeong8951@gmail.com (H.-J.A.); junpilyun@gmail.com (J.P.Y.); david.soonil.kwon@gmail.com (S.K.); seil@snu.ac.kr (S.O.); 2 Department of Internal Medicine, Seoul National University College of Medicine, Seoul 03080, Korea; gregory.lip@liverpool.ac.uk; 3 Department of Statistics and Actuarial Science, Soongsil University, Seoul 06978, Korea; hkd917@naver.com; 4 Department of Medical Statistics, College of Medicine, Catholic University of Korea, Seoul 06591, Korea; jungjin115@naver.com; 5 Liverpool Centre for Cardiovascular Science, University of Liverpool and Liverpool Chest & Heart Hospital, Liverpool L14 3PE, UK; 6 Department of Clinical Medicine, Aalborg University, 9100 Aalborg, Denmark

**Keywords:** atrial fibrillation, physical activity, dementia, prevention, cohort study, nationwide study

## Abstract

Background: It is unclear whether exercise would reduce dementia in patients with a new diagnosis of atrial fibrillation (AF). Therefore, we aimed to evaluate the association between the change in physical activity (PA) before and after new-onset AF and the risk of incident dementia. Methods: Using the Korean National Health Insurance Service database, we enrolled a total of 126,555 patients with newly diagnosed AF between 2010 and 2016, who underwent health examinations within two years before and after their diagnosis of AF. The patients were divided into four groups: persistent non-exercisers, exercise starters, exercise quitters, and exercise maintainers. Results: Based on a total of 396,503 person-years of follow-up, 5943 patients were diagnosed with dementia. Compared to persistent non-exercisers, exercise starters (adjusted hazard ratio (aHR) 0.87; 95% confidence interval (CI) 0.81–0.94), and exercise maintainers (aHR 0.66; 95% CI 0.61–0.72) showed a lower risk of incident dementia; however, the risk was similar in exercise quitters (aHR 0.98; 95% CI 0.92–1.05) (*p*-trend < 0.001). There was a J-shaped relationship between the dose of exercise and the risk of dementia, with the risk reduction maximized at 5–6 times per week of moderate-to-vigorous PA among exercise starters. Conclusion: Patients who initiated or continued regular exercise after diagnosis of AF were associated with a lower risk of dementia than persistent non-exercisers, with no risk reduction associated with exercise cessation. Our findings may provide evidence for the benefit of exercise prescription to patients with new-onset AF to prevent incident dementia regardless of their current exercise status.

## 1. Introduction

The prevalence of atrial fibrillation (AF) is rapidly increasing with an aging population, affecting 33.5 million people globally [1]. The lifetime prevalence in middle-aged adults is reported to be 25% [2]. Numerous studies have found AF to be an independent risk factor for dementia, [3] which is also a major public health issue worldwide. Furthermore, dementia is associated with an increased risk of mortality in patients with AF [4]. Hence, interventions to prevent dementia in patients with AF are needed; however, limited data on lifestyle interventions are available.

Physical activity (PA) has been proposed by the WHO to reduce the risk of dementia in the general population [5]. However, recent prospective cohort studies and randomized controlled trials have shown controversial results [6,7,8,9,10]. Since patients with AF are prevalent, and they have a two-fold increased risk of developing cognitive decline and dementia [11], this population should be considered separately from healthy controls. Previous studies regarding exercise in patients with AF mostly focused on the improvement of AF-related symptoms, quality of life, or exercise capacity [12,13,14]. AF burden was also found to decrease with regular PA [14,15]; however, there is a paucity of data on whether decreased AF burden resulting from PA could reduce incident dementia.

At the time that patients are newly diagnosed with AF, intensive lifestyle modification could be a prescription for better clinical outcomes, including the prevention of incident dementia. Based on a nationwide population-based cohort, we investigated the association between the change in PA and incident dementia among patients newly diagnosed with AF.

## 2. Materials and Methods

### 2.1. Data Source and Study Population

We collected data from the Korean National Health Insurance Service (NHIS) database from 1 January 2010 to 31 December 2016. The NHIS is a single Korean insurer covering the entire Korean population [16], and the NHIS database contains all medical claims data of the enrollees, including demographic variables, diagnosis based on International Classification of Diseases-10th Revision (ICD-10) codes, and prescription records. In addition, NHIS enrollees are recommended to receive health examinations biennially; moreover, comprehensive data from health examinations, including anthropometric measurements, laboratory findings, and answers from a self-reported questionnaire about lifestyle behavior, are added to the NHIS database. The NHIS cohort database has been used in numerous high-quality research papers [17,18], and further details about the NHIS cohort and its validity are available in a previous study [16]. The Institutional Review Board at the Seoul National University Hospital (E-2001-110-1096) approved this study, and the need for informed consent was waived since the NHIS provides anonymized data of its enrollees and strictly follows the guidelines of the Personal Data Protection Act.

**Figure 1:**
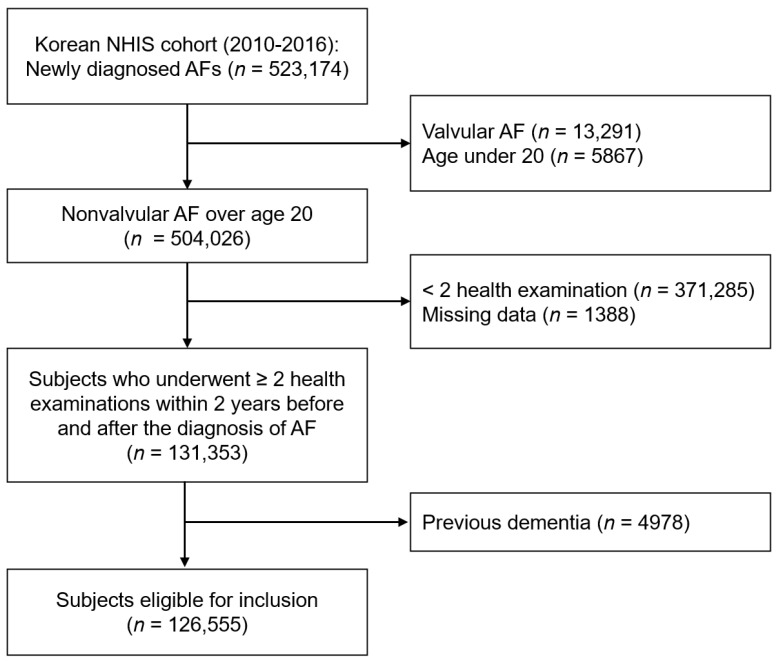
The flowchart of the study population enrollment. AF, atrial fibrillation; NHIS, National Health Insurance Service.

The flowchart of the study population enrollment is presented in Figure 1. Among the 523,174 patients newly diagnosed with AF between 2010 and 2016, patients with valvular AF and aged under 20 years were excluded. We only included patients who underwent health examinations within two years before and after their AF diagnosis and excluded those with preexisting dementia. Finally, 126,555 patients were enrolled in the analysis.

### 2.2. Evaluation of Physical Activity

The level of PA was assessed based on a self-reported structured questionnaire, which was utilized in several high-quality research papers [17,19]. The questionnaire documents the frequencies of weekly PA of varying intensities classified as light, moderate, or vigorous (Figure S1 in Supplementary Materials). Light-intensity PA was defined as walking at a usual pace for a total of 30 min/day. Moderate-intensity PA (MPA) was defined as an exercise that causes mild shortness of breath, such as brisk walking or bicycling, for a total of 30 min/day. Vigorous-intensity PA (VPA) was defined as intense exercise causing more shortness of breath than MPA, such as running, hiking, or bicycling at high speeds, for a total of 20 min/day.

According to recent European and American guidelines on the prevention of cardiovascular disease [20,21], we defined moderate to vigorous-intensity PA (MVPA) as performing regular exercise. Thus, individuals performing at least 1 regular MVPA/week were defined as an exerciser, and those with no MVPA/week were considered as a non-exerciser. For the primary analysis, the study population was categorized into four groups based on their consistency in performing regular exercise before and after AF diagnosis: [1] persistent non-exercisers, [2] exercise starters, [3] exercise quitters, and [4] exercise maintainers.

### 2.3. Covariates

We included the patients’ demographic variables, anthropometric measurements, comorbidities, medications, and health habits regarding drinking and smoking as covariates. Demographic variables of age and sex and anthropometric measurements of body mass index were included. Comorbidities included hypertension, diabetes mellitus, dyslipidemia, congestive heart failure, peripheral artery disease (PAD), previous myocardial infarction (MI), previous stroke, chronic obstructive pulmonary disease (COPD), chronic kidney disease (CKD), cancer, and CHA_2_DS_2_-VASc score. The use of medications, including oral anticoagulants (OACs), antiplatelet agents, and statins, were included. Low-income levels, smoking status, and drinking habits were also used. Detailed information about covariates is summarized in Table S1.

### 2.4. Study Outcomes and Follow-Up

The primary outcome was incident dementia. Dementia was further classified into Alzheimer’s dementia, vascular dementia, and other types of dementia. The definition of each dementia is presented in Table S1. The date of the second health examination was defined as an index date, and patients were followed up until the occurrence of the primary outcome or 31 December 2016, whichever came first (Figure S2).

### 2.5. Statistical Analysis

Baseline characteristics of patients were calculated using n (%) for categorical variables and mean ± standard deviation (SD) for continuous variables. The incidence rate (IR) was calculated based on incidence per 1000 person-years (PY), and hazard ratio (HR) and 95% confidence intervals (95% CI) for the risk of incident dementia were calculated using the Cox proportional hazards model. We used the persistent non-exerciser group as the reference group to calculate the HR of incident dementia among different PA groups. Multivariable regression models were constructed with adjustment for the following variables: (i) age and sex and (ii) age, sex, body mass index, smoking habits, drinking habits, income levels, hypertension, diabetes mellitus, dyslipidemia, congestive heart failure, PAD, previous MI, previous stroke, COPD, CKD, cancer, CHA_2_DS_2_-VASc score, and the use of OACs, antiplatelet agents, and statins.

We conducted an additional landmark analysis for two reasons. First, to minimize the possibility of reversed-causality since dementia is well known to have a long preclinical phase, and being physically inactive can be an early manifestation of dementia [22]. Second, we needed to determine whether continuing exercise for a long period could affect the development of dementia. In the landmark analysis, we excluded patients who developed incident dementia between the index date and the landmark time point. Two landmark time points of one and three years were used.

For subgroup analyses, we stratified the study population by sex, age group (<65, 65–74, and ≥75 years), and CHA_2_DS_2_-VASc score (<3 and ≥3 points). Subgroup analyses based on the presence of a history of stroke, the use of OACs, and the use of statins were also conducted since these factors are associated with the development of dementia in patients with AF [23,24,25]. *p*-for-trend and *p*-for-interaction were computed to find the intragroup and intergroup differences. To identify the optimal dose of PA for a lower risk of incident dementia, we further stratified the exercise starter group into four subgroups by the frequency of MVPA/week, which is a summation of the days of weekly MPA and VPA: 1-2/week, 3–4/week, 5–6/week, and ≥7/week. We used the same multivariable Cox regression models used in the primary analysis. All analyses were two-tailed, and statistical significance was defined as a *p*-value < 0.05. Statistical analyses were performed using SAS 9.4 (SAS Institute, Cary, NC, USA). The data underlying this article will be shared on reasonable request to the corresponding author.

## 3. Results

### 3.1. Baseline Characteristics

A total of 126,555 patients were included in this study. The baseline characteristics of each group are shown in Table 1.

Exercise quitters performed a median of 4 MVPAs/week before their AF diagnosis (interquartile range (IQR), 2–6 MVPAs/week), while exercise starters initiated a median of 4 MVPAs/week after AF diagnosis (IQR, 2–6 MVPAs/week). Exercise maintainers also performed a median of 4 MVPAs/week before and after their diagnosis of AF (IQR, 2–7 and 3–7 MVPAs/week, respectively).

### 3.2. Changes in PA before and after New AF Diagnosis and the Risk of Incident Dementia

During a mean follow-up of 3.1 ± 1.9 years (total 396,503 PY), 5943 patients were newly diagnosed with dementia (IR 15.0/1000 PY). Among 5943 patients, 4410 were diagnosed with Alzheimer’s dementia, and 951 were diagnosed with vascular dementia (IR 11.1/1000 PY and 2.4/1000 PY, respectively).

**Figure 2:**
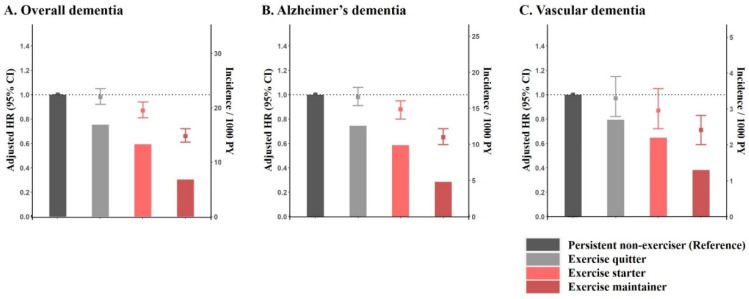
The association between change of physical activity and incident dementia in patients with newly diagnosed atrial fibrillation. Incidence/1000 person-years and adjusted hazard ratio for overall dementia (**A**), Alzheimer’s dementia (**B**), and vascular dementia (**C**) are presented in bars and error-bars, respectively.

Compared with persistent non-exercisers, exercise maintainers were associated with a lower risk of overall dementia, Alzheimer’s dementia, and vascular dementia. Exercise starters also showed lower risks; however, there were no differences in the risk of dementia between persistent non-exercisers and exercise quitters (Figure 2). Crude event numbers, IRs, and HRs for overall dementia, Alzheimer’s dementia, and vascular dementia are presented in Table S2.

### 3.3. Sensitivity Analyses: One-Year and Three-Year Landmark Analysis

Sensitivity analyses showed consistent findings with the main results (Table 2). In both the one-year and three-year landmark analyses, the risk of incident dementia of exercise quitters was similar to that of persistent non-exercisers. However, the risk of exercise starters and exercise maintainers were lower than those of persistent non-exercisers in both landmark analyses.

### 3.4. Subgroup Analyses

**Figure 3:**
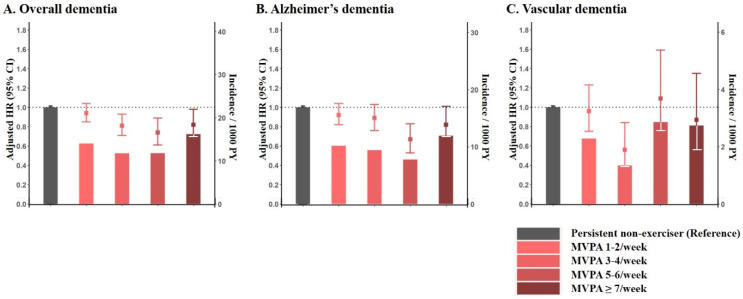
Risk of incident dementia stratified by dose of physical activity among exercise starters. Incidence/1000 person-years and adjusted hazard ratio for overall dementia (**A**), Alzheimer’s dementia (**B**), and vascular dementia (**C**) are presented in bars and error-bars, respectively. MVPA: Moderate to vigorous physical activity.

(a) Sex: There was no significant interaction between sex and change of PA in the risk of overall dementia and Alzheimer’s dementia except for vascular dementia (Table S3). In the male subgroup, exercise maintainers showed a lower risk of vascular dementia (*p*-for-trend < 0.001), which was largely in line with the main results. However, there was no significant difference in the risk of vascular dementia in the female subgroup based on the PA groups (*p*-for-trend = 0.165).

(b) Age (<65, 65–74, and ≥75 years): In all age subgroups, consistent findings with the primary analysis were observed (Table S4). There were significant interactions between age and the risk of incident dementia based on PA groups (*p*-for-interaction = 0.006), and the beneficial effects of starting and maintaining exercise after AF diagnosis were more accentuated in patients aged <65 years. 

(c) CHA_2_DS_2_-VASc score (<3 and ≥3): There were significant interactions between CHA_2_DS_2_-VASc scores of <3 and ≥3 and the risk of incident dementia based on PA groups (*p*-for-interaction = 0.008). The positive effect of exercise maintenance was more accentuated in patients with CHA_2_DS_2_-VASc scores of <3 (Table S5). In the subgroup of CHA_2_DS_2_-VASc scores of <3, exercise starters did not show a lower risk of overall dementia, possibly due to a lower incidence of dementia in this subgroup.

(d) History of stroke: The incidence rate of overall dementia was significantly higher in patients with a history of stroke. Although *p*-for-interaction between the presence of prior stroke and the exercise groups was statistically significant (*p*-for-interaction = 0.048), the exercise starters and maintainers were associated with lower risks of incident dementia regardless of the presence of a previous history of stroke (Table S6).

(e) Use of OACs: There was no significant interaction between the PA groups and the use of OACs (Table S7). However, the risk reduction of incident dementia through starting or maintaining exercise was slightly attenuated in the subgroup treated with OACs compared with those who were not on OACs.

(f) Use of statins: There was no significant interaction between PA groups and the use of statins (Table S8).

(g) Optimal dose of physical activity in exercise starters: Overall, the association between the dose of MVPA/week and the risk of dementia showed a J-shaped relationship: those with MVPAs of 5–6/week were associated with the lowest risk for developing dementia (adjusted HR 0.74, 95% CI 0.61–0.89). For Alzheimer’s dementia, the results were consistent with overall dementia. For the risk of vascular dementia, those with MVPAs of 3–4/week (adjusted HR 0.56, 95% CI 0.3–0.84) were associated with the lowest risk (Figure 3 and Table S9).

## 4. Discussion

In this large nationwide, population-based cohort study, we demonstrated that starting and maintaining exercise after AF diagnosis is associated with a significantly lower risk of incident dementia. It is also important to note that exercise quitters had a similar risk of incident dementia as persistent non-exercisers, further emphasizing the importance of exercise prescription after a new diagnosis of AF for both non-exercisers and current-exercisers. To the best of our knowledge, this is the first evidence of the beneficial effect of PA on dementia in the AF population.

At least 3 MVPAs/week were required to reduce incident dementia, and 5–6 MVPAs/week were associated with the lowest risk. These results are largely in line with guidelines for the general population provided by the European and American societies, which recommend 150 min/week of MPA or 75 min/week of VPA or an equivalent combination [20,21]. In the landmark analyses, only exercise starters showed a gradual decrease in the risk of incident dementia along with longer landmark periods. This finding suggests that once an exercise regime is initiated, an individual that continues to exercise might continuously be at a reduced risk of dementia. Meanwhile, exercise quitters showed a similar risk of incident dementia even in the landmark analyses.

In the subgroup analysis, the risk reduction was more accentuated in female exercise starters and male exercise maintainers, mainly due to significant interaction found in vascular dementia. We need careful interpretation in this finding due to a relatively large proportion of persistent non-exercisers in the female subgroup. Also, the event numbers of vascular dementia were small in both sexes. This result is in line with the previous prospective cohort study, which reported smaller relative risks of stroke in women than in men who performed the same hours of exercise although the investigators did not focus on the significance of this difference [26]. Considering the relatively less socially and culturally active lifestyle in middle-to-old aged women in Korea, they might see greater benefits from starting MVPA. However, male exercise maintainers performed more exercise than female exercise maintainers, which led to a lower risk of dementia in the male subgroup. Further study would be needed to clarify these differences. On the other hand, not only the objectively lower incidence but also the risk reduction of dementia through exercise were significant in the younger and less-diseased patients. Exercise activates neurogenesis and synaptogenesis, which could improve memory and cognitive function [27]. Thus, less significant risk reductions found in the older and more diseased patients might be attributed to their decreased synthetic function. Based on these findings, exercise should be encouraged paradoxically to the more fit and younger individuals when they are diagnosed with AF. All the subgroups stratified by prior stroke, OACs, or statins showed a consistent result with the primary outcome.

A large volume of the literature has consistently reported an increased risk of dementia in the AF population [3,11]. Considering the high prevalence and rapidly increasing incidence of AF, there is an increasing demand for interventions that can reduce the socioeconomic and health burden caused by AF. Several studies have reported that the long-term use of statins and anticoagulation therapy are associated with a lower risk of dementia in patients with AF [24,25]. Yet, the effect of lifestyle modification on the risk of dementia in patients with AF is not well reported.

Various high-quality research were conducted regarding the association between PA and dementia but were mainly on a healthy population, and even the results were conflicting; some studies have shown favorable outcomes with PA [6,7,8], while other studies obtained neutral results [9], and even greater cognitive decline was observed in patients who began to participate in the exercise program [10]. Though previous studies on healthy populations are conflicting, several plausible mechanisms could explain our findings on patients with AF.

First, the AF burden modulated by exercise might influence cognitive function. The ARIC study reported that a higher AF burden is associated with a lower cognitive function [28], and the CARDIO-FIT (Cardiorespiratory Fitness on Arrhythmia Recurrence in Obese Individuals with Atrial Fibrillation) study [14] reported that exercise significantly reduced AF burden. Furthermore, regular exercise might reduce body weight with a consequence of reduced AF burden [29]. Taken altogether, regular exercise might reduce the burden of AF with a consequent reduction in the risk of dementia. Second, patients with AF have a lower cardiac index than patients with normal sinus rhythm [30], and patients with low cardiac index are known to have an increased risk of dementia and Alzheimer’s dementia [31]. Therefore, increased cerebral blood flow owing to exercise itself [32] and improvement of left ventricular ejection fraction as a consequence of exercise in AF patients [33] might attribute to a lower risk of dementia.

### 4.1. Strengths

Our study has several strengths. First, our novel evidence was based on a large, nationwide, observational cohort. It is also important to note that we applied no strict inclusion or exclusion criteria, thereby recruiting as many patients as possible and minimizing selection bias. In addition, we collected the level of PA at two different time points before and after a new AF diagnosis, which differentiates our study from previous research [7,15] given that prior studies have only provided exercise-related information at a single time point. This is important since PA in the elderly is prone to change, resulting from illness or disability, and AF is one of the major factors that can limit exercise tolerance. Hence, collecting the exercise status at two different time points is especially important in differentiating exercise starters from persistent non-exercisers and exercise maintainers from exercise quitters. Furthermore, we added two more time points by conducting one-year and three-year landmark analyses, and the results further strengthened the primary results, minimizing the possibility of reversed-causality and highlighting the importance of starting and not quitting exercise.

### 4.2. Limitations

There are several limitations to this study. First, the study cannot show cause-and-effect relationship due to limitations inherent to the retrospective design. Second, the level of PA was evaluated by self-reported questionnaires, which inevitably accompanies recall bias. Therefore, we defined exercise as at least one regular MVPA/week, which is the most intuitive criterion that can discriminate people who intentionally exercise from those who perform only light-intensity daily activities. Third, the effect of AF diagnosis on lifestyle change could not be analyzed, so the intentionality of changes in PA could not be provided. To minimize this, we utilized two questionnaires that were closest to the date of AF diagnosis. Fourth, the exact amount of time spent on daily exercise was not available if it was over 30 min/day for MPA or 20 min/day for VPA, thereby leaving possibilities of in-group differences among exercisers. This might affect an analysis that aimed to determine the optimal frequencies of MVPA/week. In addition, a self-reported questionnaire only focuses on aerobic exercise; therefore, information on non-aerobic exercise might be missing. Fifth, the type and burden of AF were not accessible in the claims database and thus were not evaluated in the study. Lastly, due to the data characteristics, the study was conducted mainly in the Asian population; thus, further studies should be done in other population groups for results to be verified and generalized.

## 5. Conclusions

In this large, population-based cohort study, patients with new-onset AF who initiated or continued regular exercise were associated with a lower risk of incident dementia than were persistent non-exercisers. Our findings may provide evidence for physicians of exercise prescription to their patients newly diagnosed with AF, regardless of their current exercise status, to prevent or delay the onset of incident dementia.

## Figures and Tables

**Table 1:** Baseline characteristics.

Characteristics	Total (*n* = 126,555)	Non-Exerciser (*n* = 42,884)	Exercise Starter (*n* = 22,150)	Exercise Quitter (*n* = 22,993)	Exercise Maintainer (*n* = 38,528)
Age, years	62.7 ± 12.0	65.7 ± 11.5	65.2 ± 12.0	63.6 ± 11.6	59.1 ± 11.8
<65—no. (%)	64,503 (51.0%)	17,188 (40.1%)	11,689 (52.8%)	10,939 (47.6%)	24,687 (64.1%)
65–74—no. (%)	41,349 (32.7%)	15,424 (36.0%)	7181 (32.4%)	8091 (35.2%)	10,653 (27.7%)
≥75—no. (%)	20,703 (16.4%)	10,272 (24.0%)	3280 (14.8%)	3963 (17.2%)	3188 (8.3%)
Male sex (%)	78,446 (62.0%)	21,819 (50.9%)	13,537 (61.1%)	14,103 (61.3%)	28,987 (75.2%)
Comorbidities					
Hypertension	84,014 (66.4%)	29,468 (68.7%)	14,584 (65.8%)	15,432 (67.1%)	24,530 (63.7%)
Diabetes	29,024 (22.9%)	10,655 (24.9%)	5012 (22.6%)	5559 (24.2%)	7798 (20.2%)
Dyslipidemia	13,218 (10.4%)	4492 (10.5%)	2318 (10.5%)	2462 (10.7%)	3946 (10.2%)
Previous MI	6458 (5.1%)	2391 (5.6%)	1126 (5.1%)	1172 (5.1%)	1769 (4.6%)
Previous stroke	16,800 (13.3%)	6350 (14.8%)	2864 (12.9%)	3267 (14.2%)	4319 (11.2%)
Previous CHF	31,306 (24.7%)	11,896 (27.7%)	5285 (23.9%)	5904 (25.7%)	8221 (21.3%)
PAD	27,380 (21.6%)	10,552 (24.6%)	4829 (21.8%)	5318 (23.1%)	6681 (17.3%)
COPD	24,605 (19.4%)	9785 (22.8%)	4327 (19.5%)	4691 (20.4%)	5802 (15.1%)
Cancer	7253 (5.73%)	2344 (5.5%)	1363 (6.2%)	1435 (6.2%)	2111 (5.5%)
CKD	18,868 (14.9%)	7951 (18.5%)	3046 (13.8%)	3616 (15.7%)	4255 (11.0%)
CHA2DS2-VASc score	2.7 ± 1.7	3.2 ± 1.8	2.7 ± 1.7	2.9 ± 1.7	2.2 ± 1.5
0	8238 (6.5%)	1700 (4.0%)	1382 (6.2%)	1272 (5.5%)	3884 (10.1%)
1	25,590 (20.2%)	6242 (14.6%)	4537 (20.5%)	4247 (18.5%)	10,564 (27.4%)
2	28,568 (22.6%)	8498 (19.8%)	5277 (23.8%)	5122 (22.3%)	9671 (25.1%)
≥3	64,159 (50.7%)	26,444 (61.6%)	10,954 (49.5%)	12,352 (53.7%)	14,409 (37.4%)
Medication use					
OACs					
Warfarin	26,185 (20.7%)	8941 (20.9%)	4542 (20.5%)	4997 (21.7%)	7705 (20.0%)
NOACs	12,307 (9.7%)	4523 (10.6%)	2063 (9.3%)	2348 (10.2%)	3373 (8.8%)
Antiplatelet agent					
Aspirin	26,752 (21.1%)	9572 (22.3%)	4508 (20.4%)	4955 (21.6%)	7717 (20.0%)
Clopidogrel	8865 (7.0%)	3277 (7.6%)	1525 (6.9%)	1717 (7.5%)	2346 (6.1%)
Statin	23,538 (18.6%)	8466 (19.7%)	4130 (18.7%)	4367 (19.0%)	6575 (17.1%)
Anthropometric measurements					
Weight (kg)	65.3 ± 11.7	62.7 ± 11.5	65.3 ± 11.6	65.0 ± 11.5	68.3 ± 11.5
Height (cm)	162.8 ± 9.4	160.0 ± 9.5	162.8 ± 9.2	162.5 ± 9.2	166.1 ± 8.5
Waist circumference (cm)	84.6 ± 9.4	84.3 ± 10.3	84.4 ± 9.1	84.7 ± 9.0	84.8 ± 8.6
BMI (kg/m^2^)	24.6 ± 3.3	24.4 ± 3.5	24.6 ± 3.3	24.5 ± 3.3	24.7 ± 3.1
Systolic BP (mmHg)	125.7 ± 15.4	126.5 ± 16.0	125.6 ± 15.4	125.9 ± 15.7	124.9 ± 14.7
Diastolic BP (mmHg)	77.1 ± 10.3	77.1 ± 10.5	77.0 ± 10.2	77.0 ± 10.4	77.1 ± 10.1
Laboratory Findings					
Fasting glucose (mg/dL)	105.0 ± 27.3	105.4 ± 28.8	104.7 ± 27.1	105.5 ± 27.5	104.2 ± 25.6
Triglyceride (mg/dL)	132.9 ± 91.2	134.1 ± 88.5	132.6 ± 93.4	132.9 ± 88.9	131.7 ± 94.3
Total cholesterol (mg/dL)	81.0 ± 40.7	180.6 ± 40.5	181.4 ± 43.7	180.3 ± 41.4	181.8 ± 38.7
HDL-cholesterol (mg/dL)	52.2 ± 15.0	51.9 ± 14.6	52.3 ± 15.3	51.8 ± 16.2	52.7 ± 14.6
LDL-cholesterol (mg/dL)	103.2 ± 41.1	102.5 ± 38.9	103.6 ± 45.1	103.1 ± 45.7	103.7 ± 38.2
AST (mg/dL)	27.9 ± 19.0	27.8 ± 22.2	27.9 ± 16.7	27.9 ± 17.8	27.9 ± 16.9
ALT (mg/dL)	25.9 ± 20.8	24.9 ± 19.5	26.1 ± 19.4	25.8 ± 19.3	27.1 ± 23.7
GGT (mg/dL)	44.8 ± 60.7	43.3 ± 62.0	44.2 ± 58.8	45.3 ± 62.4	46.5 ± 59.4
GFR (mL/min/1.73 m^2^)	80.3 ± 28.1	78.7 ± 27.4	81.2 ± 29.7	79.9 ± 28.4	81.8 ± 27.7
Lifestyle factors					
Alcohol					
Non-drinker	84,398 (66.7%)	32,752 (76.4%)	14,644 (66.1%)	16,271 (70.8%)	20,731 (53.8%)
Mild-moderate drinker	34,842 (27.5%)	7986 (18.6%)	6272 (28.3%)	5446 (23.7%)	15,138 (39.3%)
Heavy drinker	7315 (5.8%)	2146 (5.0%)	1234 (5.6%)	1276 (5.6%)	2659 (6.9%)
Smoking					
Never smoker	75,061 (59.3%)	29,084 (67.8%)	13,092 (59.1%)	14,215 (61.8%)	18,670 (48.5%)
Ex-smoker	34,365 (27.2%)	8311 (19.4%)	5986 (27.0%)	5764 (25.1%)	14,304 (37.1%)
Current smoker	17,129 (13.5%)	5489 (12.8%)	3072 (13.9%)	3014 (13.1%)	5554 (14.4%)
Low income	21,262 (16.8%)	7876 (18.4%)	3759 (17.0%)	4018 (17.5%)	5609 (14.6%)
Follow-up duration	3.1 ± 1.9	3.1 ± 1.9	3.2 ± 1.9	3.2 ± 1.9	3.2 ± 1.9

SD, standard deviation; MI, myocardial infarction; CHF, congestive heart failure; CKD, chronic kidney disease; OACs, oral anticoagulants; NOACs, non-vitamin K antagonist oral anticoagulants; BMI, body mass index; BP, blood pressure; HDL, high-density lipoprotein; LDL, low-density lipoprotein; AST, aspartate transaminase; ALT, alanine transaminase; GGT, gamma-glutamyl transferase; GFR, glomerular filtration rate.

**Table 2:** The landmark analysis of one-year and three-year compared with the primary outcome.

Physical Activity	Primary Outcome	1-Year Landmark Analysis ^a^	3-Year Landmark Analysis ^b^
**Overall Dementia**			
Persistent non-exerciser	1 (Ref.)	1 (Ref.)	1 (Ref.)
Exercise starter	0.87 (0.81–0.94)	0.83 (0.76–0.91)	0.80 (0.70–0.91)
Exercise quitter	0.98 (0.92–1.05)	0.96 (0.89–1.04)	1.02 (0.91–1.15)
Exercise maintainer	0.66 (0.61–0.72)	0.66 (0.60–0.73)	0.65 (0.56–0.74)
	*p*-trend <0.001	*p*-trend <0.001	*p*-trend <0.001
**Alzheimer’s Dementia**			
Persistent non-exerciser	1 (Ref.)	1 (Ref.)	1 (Ref.)
Exercise starter	0.88 (0.80–0.95)	0.84 (0.76–0.93)	0.80 (0.69–0.94)
Exercise quitter	0.98 (0.91–1.06)	0.96 (0.88–1.05)	1.03 (0.90–1.18)
Exercise maintainer	0.65 (0.59–0.72)	0.65 (0.58–0.72)	0.66 (0.56–0.77)
	*p*-trend <0.001	*p*-trend <0.001	*p*-trend <0.001
**Vascular Dementia**			
Persistent non-exerciser	1 (Ref.)	1 (Ref.)	1 (Ref.)
Exercise starter	0.87 (0.72–1.05)	0.76 (0.61–0.95)	0.61 (0.43–0.88)
Exercise quitter	0.97 (0.82–1.15)	0.93 (0.77–1.13)	0.89 (0.66–1.20)
Exercise maintainer	0.71 (0.59–0.86)	0.70 (0.57–0.87)	0.56 (0.40–0.79)
	*p*-trend = 0.004	*p*-trend = 0.004	*p*-trend = 0.002

HR is adjusted for age, sex, body mass index, smoking habits, drinking habits, income level, hypertension, diabetes mellitus, dyslipidemia, previous myocardial infarction, previous stroke, previous congestive heart failure, peripheral artery disease, chronic obstructive pulmonary disease, chronic kidney disease, cancer, and the use of warfarin, non-vitamin K antagonist oral anticoagulants, aspirin, clopidogrel, and statin. ^a^ 1-year landmark analysis: excluded patients who developed dementia within 1 year after AF diagnosis. ^b^ 3-year landmark analysis: excluded patients who developed dementia within 3 years after AF diagnosis.

## Data Availability

All relevant data are within the manuscript and the supplementary material.

## Supplementary Materials

The following are available online at https://www.mdpi.com/article/10.3390/jcm10143126/s1, Figure S1: The questionnaire documenting the frequencies of weekly PA of varying intensities classified as light, moderate, or vigorous, Figure S2: Overall study flow, Table S1: Definitions of inclusion and exclusion criteria, comorbidities, lifestyle factors, and outcomes, Table S2: Risk of incident dementia by PA groups, Table S3: Risk of incident dementia by PA groups stratified by sex, Table S4: Risk of incident dementia by PA groups stratified by age, Table S5: Risk of incident dementia by PA groups stratified by CHA2DS2-VASc score, Table S6: Risk of incident dementia by PA groups stratified by history of stroke, Table S7: Risk of incident dementia by PA groups stratified by the use of oral anticoagulants (OACs), Table S8: Risk of incident dementia by PA groups stratified by the use of statins, Table S9: Association between dose of PA among exercise starter and incidence of dementia.
