# Supplementary material for: Exercise and the Risk of Dementia in Patients with Newly Diagnosed Atrial Fibrillation: A Nationwide Population-Based Study
Source: J Clin Med. 2021 Jul 15;10(14):3126. doi: 10.3390/jcm10143126 (PMC8304225; doi:10.3390/jcm10143126)
Supplement: Supplementary file 1 [file jcm-10-03126-s001.zip › jcm-1273648-supplementary.pdf]

Journal of Clinical Medicine

Supplementary Materials

Table S1. Definitions of inclusion and exclusion criteria, comorbidities, lifestyle factors, and outcomes.

Diagnosis	ICD-10-CM code and definition	Number of diagnosis
**Inclusion/Exclusion**		
Atrial fibrillation	I48.0-48.4, I48.9	Admission ≥1 or outpatient department ≥2
Valvular atrial fibrillation	I05.0, I05.2, I05.9, Z95.2-Z95.4	Admission or outpatient department ≥1
**Outcome**		
Dementia	F00-03, F107, G30, G231, G310, G311, G318 and prescription of medication for dementia, including galantamine, rivastigmine, donepezil, or memantine	Admission or outpatient department ≥1
Alzheimer's Dementia	F00, G30 and prescription of medication for dementia, including galantamine, rivastigmine, donepezil, or memantine	Admission or outpatient department ≥1
Vascular Dementia	F01 and prescription of medication for dementia, including galantamine, rivastigmine, donepezil, or memantine	Admission or outpatient department ≥1
**Based on the 2nd health examination (After AF diagnosis)**		
**Comorbidities**		
Hypertension	I10-I13, I15; and minimum 1 prescription of anti-hypertensive drug (thiazide, loop diuretics, aldosterone antagonist, alpha-/beta-blocker, calcium-channel blocker, angiotensin-converting enzyme inhibitor, angiotensin II receptor blocker) or systolic/diastolic blood pressure ≥ 140/90 mmHg	Admission ≥1 or outpatient department ≥2
Diabetes mellitus	E11-E14; and minimum 1 prescription of antidiabetic drugs (sulfonylureas, metformin, meglitinides, thiazolidinediones, dipeptidyl peptidase-4 inhibitors, α-glucosidase inhibitors, and insulin) or fasting glucose level ≥ 126 mg/dL	Admission ≥1 or outpatient department ≥2
Dyslipidemia	E78, or Total cholesterol ≥ 240 mg/dL	At least once a year
Previous MI	I21, I22	Admission or outpatient department ≥1
Previous stroke	I63, I64	Admission≥1 and brain imaging (CT or MRI) ≥1
Congestive heart failure	I50	Admission or outpatient department ≥1
PAD	I70, I73	Admission or outpatient department ≥1
COPD	J41-44	Admission ≥1
Cancer	C00-97 and RID code (V193)	Admission or outpatient department ≥1
CKD	eGFR<60ml/min/1.73m^2	
**Alcohol consumption**		
Mild to moderate drinker	Alcohol consumption > 0g to < 30g per day	
Heavy drinker	Alcohol consumption ≥ 30g per day	
**Smoking**		
Ex-smoker	Ex-smoker at the 1st examination and sustaining non-smoking till the 2nd examination	

Current smoker	Current smoker at the 2nd examination regardless of the smoking status at the 1st examination.
**Low income**	Income lowest 20% and medical aid

Abbreviation: MI, myocardial infarction; PAD, peripheral artery disease; COPD, chronic obstructive pulmonary disease; CKD, chronic kidney disease.

Table S2. Risk of incident dementia by PA groups.

Physical activity	Total number	Follow-up Duration [a]	Event number	Incidence rate [b]	Model 1[c]	Model 2[d]
**Overall Dementia**						
Persistent non-exerciser	42,884	131,555	2,949	22.4	1 (Ref.)	1 (Ref.)
Exercise starter	22,150	70,281	937	13.3	0.85 (0.79-0.92)	0.87 (0.81-0.94)
Exercise quitter	22,993	72,340	1,223	16.9	0.97 (0.91-1.04)	0.98 (0.92-1.05)
Exercise maintainer	38,528	122,328	834	6.8	0.63 (0.58-0.69)	0.66 (0.61-0.72)
					p-trend <0.001	*p*-trend <0.001
**Alzheimer's Dementia**						
Persistent non-exerciser	42,884	131,555	2,225	16.9	1 (Ref.)	1 (Ref.)
Exercise starter	22,150	70,281	692	9.9	0.86 (0.78-0.93)	0.88 (0.80-0.95)
Exercise quitter	22,993	72,340	909	12.6	0.98 (0.90-1.05)	0.98 (0.91-1.06)
Exercise maintainer	38,528	122,328	584	4.8	0.62 (0.56-0.68)	0.65 (0.59-0.72)
					p-trend <0.001	*p*-trend <0.001
**Vascular Dementia**						
Persistent non-exerciser	42,884	131,555	444	3.4	1 (Ref.)	1 (Ref.)
Exercise starter	22,150	70,281	152	2.2	0.85 (0.70-1.02)	0.87 (0.72-1.05)
Exercise quitter	22,993	72,340	194	2.7	0.96 (0.81-1.14)	0.97 (0.82-1.15)
Exercise maintainer	38,528	122,328	161	1.3	0.68 (0.57-0.82)	0.71 (0.59-0.86)
					p-trend= 0.001	*p*-trend= 0.004

[a] Follow-up duration showed as person-years; [b] Incidence rate = Event number / 1000 person-years; [c] Model 1 adjusted for age and sex; [d] Model 2 adjusted for age, sex, body mass index, smoking habits, drinking habits, income level, hypertension, diabetes mellitus, dyslipidemia, congestive heart failure, peripheral artery disease, previous myocardial infarction, previous stroke, chronic obstructive pulmonary disease, chronic kidney disease, cancer, CHA2DS2-VASc score, and the use of oral anticoagulants, antiplatelet agents, and statin.

Table S3. Risk of incident dementia by PA groups stratified by sex.

Physical activity	Total number	Follow-up Duration[a]	Event number	Incidence rate[b]	Model 1[c]	Model 2[d]
**Overall Dementia**					*p*-for-interaction= 0.158	*p*-for-interaction= 0.199
**Male**						
Persistent non-exerciser	21,819	66,372	1,197	18.0	1 (Ref.)	1 (Ref.)
Exercise starter	13,537	42,777	495	11.6	0.87 (0.78-0.97)	0.90 (0.81-1.00)
Exercise quitter	14,103	44,409	626	14.1	0.94 (0.85-1.03)	0.96 (0.87-1.06)
Exercise maintainer	28,987	92,430	537	5.8	0.59 (0.54-0.66)	0.64 (0.57-0.71)
					p-trend <0.001	*p*-trend <0.001
**Female**						
Persistent non-exerciser	21,065	65,183	1752	26.9	1 (Ref.)	1 (Ref.)
Exercise starter	8,613	27,505	442	16.1	0.83 (0.75-0.92)	0.85 (0.76-0.94)
Exercise quitter	8,890	27,931	597	21.4	1.00 (0.91-1.10)	1.00 (0.91-1.10)
Exercise maintainer	9,541	29,897	297	9.9	0.70 (0.62-0.79)	0.73 (0.64-0.82)
					p-trend <0.001	*p*-trend <0.001
**Alzheimer's Dementia**					*p*-for-interaction= 0.399	*p*-for-interaction= 0.511
**Male**						
Persistent non-exerciser	21,819	66,372	881	13.3	1 (Ref.)	1 (Ref.)
Exercise starter	13,537	42,777	351	8.2	0.86 (0.76-0.97)	0.89 (0.79-1.01)
Exercise quitter	14,103	44,409	447	10.1	0.92 (0.82-1.04)	0.95 (0.85-1.07)
Exercise maintainer	28,987	92,430	375	4.1	0.59 (0.52-0.67)	0.64 (0.56-0.72)
					p-trend <.001	*p*-trend <.001
**Female**						
Persistent non-exerciser	21,065	65,183	1344	20.6	1 (Ref.)	1 (Ref.)
Exercise starter	8,613	27,505	341	12.4	0.85 (0.75-0.95)	0.86 (0.77-0.97)
Exercise quitter	8,890	27,931	462	16.5	1.02 (0.92-1.14)	1.02 (0.92-1.14)
Exercise maintainer	9,541	29,897	209	7.0	0.66 (0.57-0.77)	0.69 (0.59-0.80)
					p-trend <0.001	*p*-trend <0.001
**Vascular Dementia**					*p*-for-interaction= 0.025	*p*-for-interaction= 0.027
**Male**						
Persistent non-exerciser	21,819	66,372	184	2.8	1 (Ref.)	1 (Ref.)
Exercise starter	13,537	42,777	91	2.1	0.96 (0.75-1.24)	0.99 (0.77-1.33)
Exercise quitter	14,103	44,409	120	2.7	1.11 (0.88-1.40)	1.13 (0.90-1.42)
Exercise maintainer	28,987	92,430	102	1.1	0.63 (0.49-0.80)	0.66 (0.52-0.85)
					p-trend <0.001	*p*-trend <0.001

**Female**						
Persistent non-exerciser	21,065	65,183	260	4.0	1 (Ref.)	1 (Ref.)
Exercise starter	8,613	27,505	61	2.2	0.73 (0.55-0.97)	0.76 (0.57-1.01)
Exercise quitter	8,890	27,931	74	2.6	0.81 (0.62-1.04)	0.82 (0.63-1.06)
Exercise maintainer	9,541	29,897	59	2.0	0.84 (0.63-1.13)	0.88 (0.67-1.17)
					p-trend= 0.091	*p*-trend= 0.165

HR, hazard ratio; CI, confidence interval

[a]Follow-up duration showed as person-years

[b]Incidence rate = Event number / 1000 person-years

[c]Model 1 adjusted for age and sex

[d]Model 2 adjusted for age, sex, body mass index, smoking habits, drinking habits, income level, hypertension, diabetes mellitus, dyslipidemia, congestive heart failure, peripheral artery disease, previous myocardial infarction, previous stroke, chronic obstructive pulmonary disease, chronic kidney disease, cancer, CHA2DS2-VASc score, and the use of oral anticoagulants, antiplatelet agents, and statin.

Table S4. Risk of incident dementia by PA groups stratified by age.

Physical activity	Total number	Follow-up Duration [a]	Event number	Incidence rate[b]	Model 1[c]	Model 2[d]
**Overall Dementia**					*p*-for-interaction= 0.008	*p*-for-interaction= 0.006
**Age <65**						
Persistent non-exerciser	17,188	57,011	238	432	1 (Ref.)	1 (Ref.)
Exercise starter	11,689	39,334	105	237	0.71 (0.56-0.89)	0.73 (0.58-0.91)
Exercise quitter	10,939	36,966	121	3.3	0.82 (0.66-1.02)	0.82 (0.66-1.02)
Exercise maintainer	24,687	80,984	126	1.6	0.48 (0.39-0.60)	0.51 (0.41-0.64)
					p-trend <0.001	*p*-trend <0.001
**Age 65-74**						
Persistent non-exerciser	15,424	48,137	1,141	23.7	1 (Ref.)	1 (Ref.)
Exercise starter	7,181	22,355	409	18.3	0.83 (0.74-0.93)	0.86 (0.77-0.96)
Exercise quitter	8,091	25,127	531	21.1	0.95 (0.86-1.05)	0.96 (0.87-1.06)
Exercise maintainer	10,653	33,161	402	12.1	0.59 (0.53-0.67)	0.63 (0.56-0.71)
					p-trend <0.001	*p*-trend <0.001
**Age ≥75**						
Persistent non-exerciser	10,272	26,407	1,570	59.5	1 (Ref.)	1 (Ref.)
Exercise starter	3,280	8,592	423	49.2	0.89 (0.80-0.99)	0.90 (0.81-1.00)
Exercise quitter	3,963	10,246	571	55.7	1.00 (0.91-1.10)	1.01 (0.92-1.11)
Exercise maintainer	3,188	8,183	306	37.4	0.74 (0.66-0.84)	0.77 (0.68-0.87)
					p-trend <0.001	*p*-trend <0.001
**Alzheimer's Dementia**					*p*-for-interaction= 0.010	*p*-for-interaction= 0.008
**Age <65**						
Persistent non-exerciser	17,188	57,011	148	2.6	1 (Ref.)	1 (Ref.)
Exercise starter	11,689	39,334	69	1.8	0.77 (0.58-1.03)	0.79 (0.60-1.06)
Exercise quitter	10,939	36,966	72	1.9	0.80 (0.61-1.07)	0.80 (0.60-1.06)
Exercise maintainer	24,687	80,984	74	0.9	0.49 (0.37-0.65)	0.52 (0.39-0.70)
					p-trend <0.001	*p*-trend <0.001
**Age 65-74**						
Persistent non-exerciser	15,424	48,137	834	17.3	1 (Ref.)	1 (Ref.)
Exercise starter	7,181	22,355	296	13.2	0.83 (0.72-0.94)	0.86 (0.75-0.98)
Exercise quitter	8,091	25,127	384	15.3	0.95 (0.84-1.07)	0.95 (0.85-1.08)
Exercise maintainer	10,653	33,161	270	8.1	0.56 (0.48-0.64)	0.59 (0.51-0.68)
					p-trend <0.001	*p*-trend <0.001
**Age ≥75**						
Persistent non-exerciser	10,272	26,407	1,243	47.1	1 (Ref.)	1 (Ref.)

Exercise starter	3,280	8,592	327	38.1	0.87 (0.77-0.99)	0.89 (0.78-1.00)
Exercise quitter	3,963	10,246	453	44.2	1.01 (0.90-1.12)	1.02 (0.92-1.14)
Exercise maintainer	3,188	8,183	240	29.3	0.75 (0.65-0.86)	0.78 (0.68-0.90)
					p-trend <0.001	*p*-trend= 0.001
**Vascular Dementia**					*p*-for-interaction= 0.264	*p*-for-interaction= 0.204
**Age <65**						
Persistent non-exerciser	17,188	57,011	64	1.1	1 (Ref.)	1 (Ref.)
Exercise starter	11,689	39,334	19	0.5	0.45 (0.27-0.75)	0.45 (0.27-0.75)
Exercise quitter	10,939	36,966	35	1.0	0.84 (0.55-1.26)	0.83 (0.55-1.25)
Exercise maintainer	24,687	80,984	38	0.5	0.47 (0.31-0.70)	0.47 (0.31-0.72)
					p-trend <0.001	*p*-trend <0.001
**Age 65-74**						
Persistent non-exerciser	15,424	48,137	184	3.8	1 (Ref.)	1 (Ref.)
Exercise starter	7,181	22,355	76	3.4	0.94 (0.72-1.23)	0.98 (0.75-1.28)
Exercise quitter	8,091	25,127	92	3.7	1.01 (0.78-1.30)	1.03 (0.80-1.32)
Exercise maintainer	10,653	33,161	83	2.5	0.74 (0.57-0.96)	0.79 (0.61-1.03)
					p-trend= 0.121	*p*-trend= 0.293
**Age ≥75**						
Persistent non-exerciser	10,272	26,407	196	7.4	1 (Ref.)	1 (Ref.)
Exercise starter	3,280	8,592	57	6.6	0.93 (0.69-1.23)	0.95 (0.71-1.28)
Exercise quitter	3,963	10,246	67	6.5	0.92 (0.70-1.22)	0.93 (0.71-1.23)
Exercise maintainer	3,188	8,183	40	4.9	0.74 (0.52-1.05)	0.75 (0.53-1.07)
					p-trend= 0.396	*p*-trend= 0.462

HR, hazard ratio; CI, confidence interval

[a]Follow-up duration showed as person-years

[b]Incidence rate = Event number / 1000 person-years

[c]Model 1 adjusted for age and sex

[d]Model 2 adjusted for age, sex, body mass index, smoking habits, drinking habits, income level, hypertension, diabetes mellitus, dyslipidemia, congestive heart failure, peripheral artery disease, previous myocardial infarction, previous stroke, chronic obstructive pulmonary disease, chronic kidney disease, cancer, CHA2DS2-VASc score, and the use of oral anticoagulants, antiplatelet agents, and statin.

Table S5. Risk of incident dementia by PA groups stratified by CHA_2DS_2-VASc score.

Physical activity	Total number	Follow-up Duration [a]	Event number	Incidence rate[b]	Model 1[c]	Model 2[d]
**Overall Dementia**					*p*-for-interaction= 0.008	*p*-for-interaction= 0.008
**CHA_2DS_2-VASc score <3**						
Persistent non-exerciser	16,440	55,193	357	6.5	1 (Ref.)	1 (Ref.)
Exercise starter	11,196	37,791	168	4.4	0.85 (0.70-1.02)	0.86 (0.72-1.04)
Exercise quitter	10,641	36,213	175	4.8	0.82 (0.68-0.98)	0.84 (0.70-1.01)
Exercise maintainer	24,119	80,071	189	2.4	0.54 (0.45-0.65)	0.56 (0.47-0.67)
					p-trend <0.001	*p*-trend <0.001
**CHA_2DS_2-VASc score ≥3**						
Persistent non-exerciser	26,444	76,362	2,592	33.9	1 (Ref.)	1 (Ref.)
Exercise starter	10,954	32,490	769	23.7	0.85 (0.78-0.92)	0.87 (0.80-0.94)
Exercise quitter	12,352	36,127	1,048	29.0	0.99 (0.92-1.07)	1.00 (0.93-1.07)
Exercise maintainer	14,409	42,256	645	15.3	0.67 (0.62-0.73)	0.70 (0.64-0.76)
					p-trend <0.001	*p*-trend <0.001
**Alzheimer's Dementia**					*p*-for-interaction= 0.082	*p*-for-interaction= 0.086
**CHA_2DS_2-VASc score <3**						
Persistent non-exerciser	16,440	55,193	239	4.3	1 (Ref.)	1 (Ref.)
Exercise starter	11,196	37,791	119	3.1	0.92 (0.74-1.15)	0.93 (0.75-1.16)
Exercise quitter	10,641	36,213	119	3.3	0.84 (0.68-1.05)	0.87 (0.70-1.08)
Exercise maintainer	24,119	80,071	126	1.6	0.56 (0.45-0.70)	0.59 (0.47-0.73)
					p-trend <0.001	*p*-trend <0.001
**CHA_2DS_2-VASc score ≥3**						
Persistent non-exerciser	26,444	76,362	1,986	26.0	1 (Ref.)	1 (Ref.)
Exercise starter	10,954	32,490	573	17.6	0.84 (0.77-0.92)	0.86 (0.78-0.94)
Exercise quitter	12,352	36,127	790	21.9	0.99 (0.91-1.08)	1.00 (0.92-1.09)
Exercise maintainer	14,409	42,256	458	10.8	0.65 (0.59-0.72)	0.67 (0.61-0.75)
					p-trend <0.001	*p*-trend <0.001
**Vascular Dementia**					*p*-for-interaction= 0.185	*p*-for-interaction= 0.154
**CHA_2DS_2-VASc score <3**						
Persistent non-exerciser	16,440	55,193	71	1.3	1 (Ref.)	1 (Ref.)
Exercise starter	11,196	37,791	31	0.8	0.73 (0.48-1.12)	0.75 (0.49-1.14)
Exercise quitter	10,641	36,213	39	1.1	0.88 (0.59-1.30)	0.90 (0.61-1.33)

Exercise maintainer	24,119	80,071	40	0.5	0.51 (0.34-0.75)	0.52 (0.35-0.77)
					p-trend= 0.007	p-trend= 0.011
**CHA₂DS₂-VASc score ≥3**						
Persistent non-exerciser	26,444	76,362	373	4.9	1 (Ref.)	1 (Ref.)
Exercise starter	10,954	32,490	121	3.7	0.87 (0.71-1.07)	0.89 (0.73-1.10)
Exercise quitter	12,352	36,127	155	4.3	0.97 (0.80-1.17)	0.98 (0.81-1.18)
Exercise maintainer	14,409	42,256	121	2.9	0.77 (0.62-0.95)	0.79 (0.64-0.97)
					p-trend= 0.082	p-trend= 0.141

HR, hazard ratio; CI, confidence interval

[a]Follow-up duration showed as person-years

[b]Incidence rate = Event number / 1000 person-years

[c]Model 1 adjusted for age and sex

[d]Model 2 adjusted for age, sex, body mass index, smoking habits, drinking habits, income level, hypertension, diabetes mellitus, dyslipidemia, congestive heart failure, peripheral artery disease, previous myocardial infarction, previous stroke, chronic obstructive pulmonary disease, chronic kidney disease, cancer, CHA2DS2-VASc score, and the use of oral anticoagulants, antiplatelet agents, and statin.

Table S6. Risk of incident dementia by PA groups stratified by history of stroke.

Physical activity	Total number	Follow-up Duration [a]	Event number	Incidence rate[b]	Model 1[c]	Model 2[d]
**Overall Dementia**					*p*-for-interaction= 0.061	*p*-for-interaction= 0.048
**No prior stroke**						
Persistent non-exerciser	36,534	113,647	2,181	19.2	1 (Ref.)	1 (Ref.)
Exercise starter	19,286	61,773	706	11.4	0.87 (0.80-0.95)	0.88 (0.81-0.96)
Exercise quitter	19,726	62,883	898	14.3	0.98 (0.90-1.06)	0.99 (0.92-1.07)
Exercise maintainer	34,209	109,636	611	5.6	0.64 (0.58-0.70)	0.66 (0.61-0.73)
					p-trend <0.001	*p*-trend <0.001
**Prior stroke**						
Persistent non-exerciser	6,350	17,909	768	42.9	1 (Ref.)	1 (Ref.)
Exercise starter	2,864	8,508	231	27.1	0.82 (0.71-0.95)	0.83 (0.72-0.96)
Exercise quitter	3,267	9,457	325	34.4	0.95 (0.83-1.08)	0.95 (0.83-1.08)
Exercise maintainer	4,319	12,692	223	17.6	0.64 (0.55-0.75)	0.67 (0.57-0.78)
					p-trend <0.001	*p*-trend <0.001
**Alzheimer's Dementia**					*p*-for-interaction= 0.168	*p*-for-interaction= 0.144
**No prior stroke**						
Persistent non-exerciser	36,534	113,647	1,666	14.7	1 (Ref.)	1 (Ref.)
Exercise starter	19,286	61,773	526	8.5	0.87 (0.79-0.96)	0.88 (0.80-0.97)
Exercise quitter	19,726	62,883	676	10.8	0.98 (0.90-1.08)	0.99 (0.91-1.09)
Exercise maintainer	34,209	109,636	433	3.9	0.62 (0.56-0.70)	0.65 (0.58-0.72)
					p-trend <0.001	*p*-trend <0.001
**Prior stroke**						
Persistent non-exerciser	6,350	17,909	559	31.2	1 (Ref.)	1 (Ref.)
Exercise starter	2,864	8,508	166	19.5	0.83 (0.70-0.99)	0.84 (0.71-1.00)
Exercise quitter	3,267	9,457	233	24.6	0.95 (0.82-1.11)	0.96 (0.82-1.12)
Exercise maintainer	4,319	12,692	151	11.9	0.64 (0.53-0.76)	0.66 (0.55-0.79)
					p-trend <0.001	*p*-trend <0.001
**Vascular Dementia**					*p*-for-interaction= 0.354	*p*-for-interaction= 0.341
**No prior stroke**						
Persistent non-exerciser	36,534	113,647	302	2.7	1 (Ref.)	1 (Ref.)
Exercise starter	19,286	61,773	111	1.8	0.91 (0.73-1.13)	0.92 (0.74-1.15)
Exercise quitter	19,726	62,883	133	2.1	0.98 (0.80-1.21)	1.00 (0.81-1.22)

Exercise maintainer	34,209	109,636	107	1.0	0.68 (0.54-0.86)	0.70 (0.55-0.88)
					p-trend= 0.009	*p*-trend= 0.016
**Prior stroke**						
Persistent non-exerciser	6,350	17,909	142	7.9	1 (Ref.)	1 (Ref.)
Exercise starter	2,864	8,508	41	4.8	0.73 (0.51-1.04)	0.74 (0.52-1.05)
Exercise quitter	3,267	9,457	61	6.5	0.91 (0.67-1.23)	0.91 (0.67-1.24)
Exercise maintainer	4,319	12,692	54	4.3	0.73 (0.53-1.01)	0.75 (0.54-1.04)
					p-trend= 0.149	*p*-trend= 0.203

HR, hazard ratio; CI, confidence interval

[a]Follow-up duration showed as person-years

[b]Incidence rate = Event number / 1000 person-years

[c]Model 1 adjusted for age and sex

[d]Model 2 adjusted for age, sex, body mass index, smoking habits, drinking habits, income level, hypertension, diabetes mellitus, dyslipidemia, congestive heart failure, peripheral artery disease, previous myocardial infarction, previous stroke, chronic obstructive pulmonary disease, chronic kidney disease, cancer, CHA2DS2-VASc score, and the use of oral anticoagulants, antiplatelet agents, and statin.

Table S7. Risk of incident dementia by PA groups stratified by the use of oral anticoagulants (OACs).

Physical activity	Total number	Follow-up Duration [a]	Event number	Incidence rate[b]	Model 1[c]	Model 2[d]
**Overall Dementia**					*p*-for-interaction= 0.114	*p*-for-interaction= 0.162
**Patients not on OACs**						
Persistent non-exerciser	30,549	99,510	2,109	21.2	1 (Ref.)	1 (Ref.)
Exercise starter	16,024	53,529	658	12.3	0.84 (0.77-0.92)	0.85 (0.78-0.93)
Exercise quitter	16,245	54,158	859	15.9	0.97 (0.90-1.05)	0.99 (0.91-1.07)
Exercise maintainer	28,242	93,899	558	5.9	0.61 (0.55-0.67)	0.64 (0.58-0.70)
					p-trend <0.001	*p*-trend <0.001
**Patients on OACs**						
Persistent non-exerciser	12,335	32,045	840	26.2	1 (Ref.)	1 (Ref.)
Exercise starter	6,126	16,752	279	16.7	0.89 (0.77-1.02)	0.92 (0.80-1.05)
Exercise quitter	6,748	18,182	364	20.0	0.96 (0.85-1.09)	0.97 (0.85-1.09)
Exercise maintainer	10,286	28,429	276	9.7	0.69 (0.60-0.80)	0.73 (0.64-0.84)
					p-trend <0.001	*p*-trend <0.001
**Alzheimer's Dementia**					*p*-for-interaction= 0.506	*p*-for-interaction= 0.587
**Patients not on OACs**						
Persistent non-exerciser	30,549	99,510	1,583	15.9	1 (Ref.)	1 (Ref.)
Exercise starter	16,024	53,529	497	9.3	0.86 (0.78-0.95)	0.88 (0.79-0.97)
Exercise quitter	16,245	54,158	642	11.9	0.99 (0.90-1.08)	1.00 (0.91-1.10)
Exercise maintainer	28,242	93,899	395	4.2	0.60 (0.54-0.67)	0.63 (0.56-0.71)
					p-trend <0.001	*p*-trend <0.001
**Patients on OACs**						
Persistent non-exerciser	12,335	32,045	642	20.0	1 (Ref.)	1 (Ref.)
Exercise starter	6,126	16,752	195	11.6	0.84 (0.71-0.99)	0.87 (0.74-1.02)
Exercise quitter	6,748	18,182	267	14.7	0.95 (0.82-1.09)	0.95 (0.83-1.10)
Exercise maintainer	10,286	28,429	189	6.6	0.66 (0.56-0.79)	0.70 (0.59-0.83)
					p-trend <0.001	*p*-trend <0.001
**Vascular Dementia**					*p*-for-interaction= 0.223	*p*-for-interaction= 0.252
**Patients not on OACs**						
Persistent non-exerciser	30,549	99,510	316	3.2	1 (Ref.)	1 (Ref.)
Exercise starter	16,024	53,529	100	1.9	0.79 (0.63-0.99)	0.81 (0.64-1.01)
Exercise quitter	16,245	54,158	133	2.5	0.95 (0.78-1.17)	0.96 (0.78-1.18)

Exercise maintainer	28,242	93,899	102	1.1	0.63 (0.50-0.80)	0.66 (0.52-0.83)
					p-trend <0.001	*p*-trend= 0.003
**Patients on OACs**						
Persistent non-exerciser	12,335	32,045	128	4.0	1 (Ref.)	1 (Ref.)
Exercise starter	6,126	16,752	52	3.1	0.98 (0.71-1.35)	1.01 (0.73-1.40)
Exercise quitter	6,748	18,182	61	3.4	0.97 (0.72-1.32)	0.99 (0.73-1.34)
Exercise maintainer	10,286	28,429	59	2.1	0.79 (0.57-1.09)	0.84 (0.61-1.16)
					p-trend= 0.522	*p*-trend= 0.702

HR, hazard ratio; CI, confidence interval

[a]Follow-up duration showed as person-years

[b]Incidence rate = Event number / 1000 person-years

[c]Model 1 adjusted for age and sex

[d]Model 2 adjusted for age, sex, body mass index, smoking habits, drinking habits, income level, hypertension, diabetes mellitus, dyslipidemia, congestive heart failure, peripheral artery disease, previous myocardial infarction, previous stroke, chronic obstructive pulmonary disease, chronic kidney disease, cancer, CHA2DS2-VASc score, and the use of oral anticoagulants, antiplatelet agents, and statin.

Table S8. Risk of incident dementia by PA groups stratified by the use of statins.

Physical activity	Total number	Follow-up Duration [a]	Event number	Incidence rate[b]	Model 1[c]	Model 2[d]
**Overall Dementia**					*p*-for-interaction= 0.128	*p*-for-interaction= 0.145
**Patients not on statins**						
Persistent non-exerciser	34,418	107,115	2,320	21.7	1 (Ref.)	1 (Ref.)
Exercise starter	18,020	57,969	758	13.1	0.88 (0.81-0.95)	0.90 (0.83-0.98)
Exercise quitter	18,626	59,395	979	16.5	0.99 (0.92-1.07)	1.00 (0.93-1.08)
Exercise maintainer	31,953	102,748	641	6.2	0.62 (0.57-0.68)	0.66 (0.60-0.72)
					p-trend <0.001	*p*-trend <0.001
**Patients on statins**						
Persistent non-exerciser	8,466	24,440	629	25.7	1 (Ref.)	1 (Ref.)
Exercise starter	4,130	12,312	179	14.5	0.75 (0.63-0.89)	0.77 (0.65-0.91)
Exercise quitter	4,367	12,945	244	18.8	0.90 (0.78-1.04)	0.91 (0.79-1.06)
Exercise maintainer	6,575	19,580	193	9.9	0.66 (0.56-0.78)	0.70 (0.59-0.83)
					p-trend <0.001	*p*-trend <0.001
**Alzheimer's Dementia**					*p*-for-interaction= 0.416	*p*-for-interaction= 0.426
**Patients not on statins**						
Persistent non-exerciser	34,418	107,115	1,743	16.3	1 (Ref.)	1 (Ref.)
Exercise starter	18,020	57,969	557	9.6	0.88 (0.80-0.97)	0.90 (0.82-0.99)
Exercise quitter	18,626	59,395	729	12.3	1.00 (0.92-1.09)	1.01 (0.93-1.10)
Exercise maintainer	31,953	102,748	452	4.4	0.62 (0.56-0.69)	0.65 (0.58-0.72)
					p-trend <0.001	*p*-trend <0.001
**Patients on statins**						
Persistent non-exerciser	8,466	24,440	482	19.7	1 (Ref.)	1 (Ref.)
Exercise starter	4,130	12,312	135	11.0	0.76 (0.63-0.92)	0.78 (0.64-0.94)
Exercise quitter	4,367	12,945	180	13.9	0.89 (0.75-1.05)	0.90 (0.76-1.07)
Exercise maintainer	6,575	19,580	132	6.7	0.63 (0.51-0.76)	0.66 (0.54-0.81)
					p-trend <0.001	*p*-trend <0.001
**Vascular Dementia**					*p*-for-interaction= 0.010	*p*-for-interaction= 0.102
**Patients not on statins**						
Persistent non-exerciser	34,418	107,115	345	3.2	1 (Ref.)	1 (Ref.)
Exercise starter	18,020	57,969	126	2.2	0.91 (0.74-1.12)	0.94 (0.76-1.15)
Exercise quitter	18,626	59,395	153	2.6	0.98 (0.81-1.19)	0.99 (0.82-1.20)

Exercise maintainer	31,953	102,748	116	1.1	0.65 (0.52-0.80)	0.68 (0.54-0.84)
					p-trend <0.001	*p*-trend= 0.004
**Patients on statins**						
Persistent non-exerciser	8,466	24,440	99	4.1	1 (Ref.)	1 (Ref.)
Exercise starter	4,130	12,312	26	2.1	0.63 (0.41-0.97)	0.64 (0.41-0.99)
Exercise quitter	4,367	12,945	41	3.2	0.88 (0.61-1.28)	0.89 (0.62-1.29)
Exercise maintainer	6,575	19,580	45	2.3	0.80 (0.56-1.16)	0.84 (0.58-1.21)
					p-trend= 0.181	*p*-trend= 0.236

HR, hazard ratio; CI, confidence interval

[a]Follow-up duration showed as person-years

[b]Incidence rate = Event number / 1000 person-years

[c]Model 1 adjusted for age and sex

[d]Model 2 adjusted for age, sex, body mass index, smoking habits, drinking habits, income level, hypertension, diabetes mellitus, dyslipidemia, congestive heart failure, peripheral artery disease, previous myocardial infarction, previous stroke, chronic obstructive pulmonary disease, chronic kidney disease, cancer, CHA2DS2-VASc score, and the use of oral anticoagulants, antiplatelet agents, and statin.

Table S9. Association between dose of PA among exercise starter and incidence of dementia.

Physical activity	Total number	Follow-up duration[a]	Event number	Incidence rate [b]	Model 1[c]	Model 2[d]
**Overall Dementia**						
Persistent non-exerciser	42,884	131,555	2,949	22.4	1 (Ref.)	1 (Ref.)
MVPA 1-2/week	10,307	32,803	460	14.0	0.92 (0.84-1.02)	0.94 (0.85-1.04)
MVPA 3-4/week	6,085	19,292	227	11.8	0.78 (0.68-0.89)	0.81 (0.71-0.93)
MVPA 5-6/week	3,195	10,156	120	11.8	0.73 (0.61-0.88)	0.74 (0.61-0.89)
MVPA ≥7/week	2,563	8,030	130	16.2	0.79 (0.66-0.94)	0.82 (0.69-0.98)
					p-trend <0.001	*p*-trend <0.001
**Alzheimer's Dementia**						
Persistent non-exerciser	42,884	131,555	2,225	16.9	1 (Ref.)	1 (Ref.)
MVPA 1-2/week	10,307	32,803	335	10.2	0.91 (0.81-1.02)	0.92 (0.82-1.04)
MVPA 3-4/week	6,085	19,292	182	9.4	0.86 (0.74-1.00)	0.89 (0.76-1.03)
MVPA 5-6/week	3,195	10,156	79	7.8	0.66 (0.53-0.83)	0.67 (0.53-0.83)
MVPA ≥7/week	2,563	8,030	96	12.0	0.79 (0.64-0.97)	0.82 (0.67-1.01)
					p-trend <0.001	*p*-trend= 0.001
**Vascular Dementia**						
Persistent non-exerciser	42,884	131,555	444	3.4	1 (Ref.)	1 (Ref.)
MVPA 1-2/week	10,307	32,803	75	2.3	0.94 (0.73-1.20)	0.96 (0.75-1.23)
MVPA 3-4/week	6,085	19,292	26	1.3	0.54 (0.36-0.80)	0.56 (0.38-0.84)
MVPA 5-6/week	3,195	10,156	29	2.9	1.08 (0.74-1.57)	1.09 (0.75-1.59)
MVPA ≥7/week	2,563	8,030	22	2.7	0.85 (0.55-1.30)	0.88 (0.547-1.35)
					p-trend =0.040	*p*-trend =0.069

[a]Follow-up duration showed as person-years

[b]Incidence rate = Event number / 1000 person-years

[c]Model 1 adjusted for age and sex

[d]Model 2 adjusted for age, sex, body mass index, smoking habits, drinking habits, income level, hypertension, diabetes mellitus, dyslipidemia, congestive heart failure, peripheral artery disease, previous myocardial infarction, previous stroke, chronic obstructive pulmonary disease, chronic kidney disease, cancer, CHA2DS2-VASc score, and the use of oral anticoagulants, antiplatelet agents, and statin

Supplementary Figures

1. Read the questions below and **mark the box that most corresponds to your physical activity status in the past week**.

1-1. In the last week, how many days did you perform more than 20 minutes a day of intense activity that made your breath much shorter than usual?

(Examples: running, aerobic, bicycling at high speed, hiking, etc.)

☐ none ☐ 1 day ☐ 2 days ☐ 3 days ☐ 4 days ☐ 5 days ☐ 6 days ☐ 7 days

1-2. In the last week, how many days did you perform a moderate activity that made your breath a little shorter than usual? (※ **Should exclude activities that are related to question 1-1**)

(Examples: brisk walking, doubles tennis, bicycling at normal speed, mopping, etc.)

☐ none ☐ 1 day ☐ 2 days ☐ 3 days ☐ 4 days ☐ 5 days ☐ 6 days ☐ 7 days

1-3. In the last week, how many days did you walk for more than 30 minutes a day, totaling at least 10 minutes at a time? (※ **Should exclude activities that are related to question 1-1 and 1-2**)

(Examples: light exercise, including walking in commuting or leisure time)

☐ none ☐ 1 day ☐ 2 days ☐ 3 days ☐ 4 days ☐ 5 days ☐ 6 days ☐ 7 days

Figure S1. The questionnaire documenting the frequencies of weekly PA of varying intensities classified as light, moderate, or vigorous.

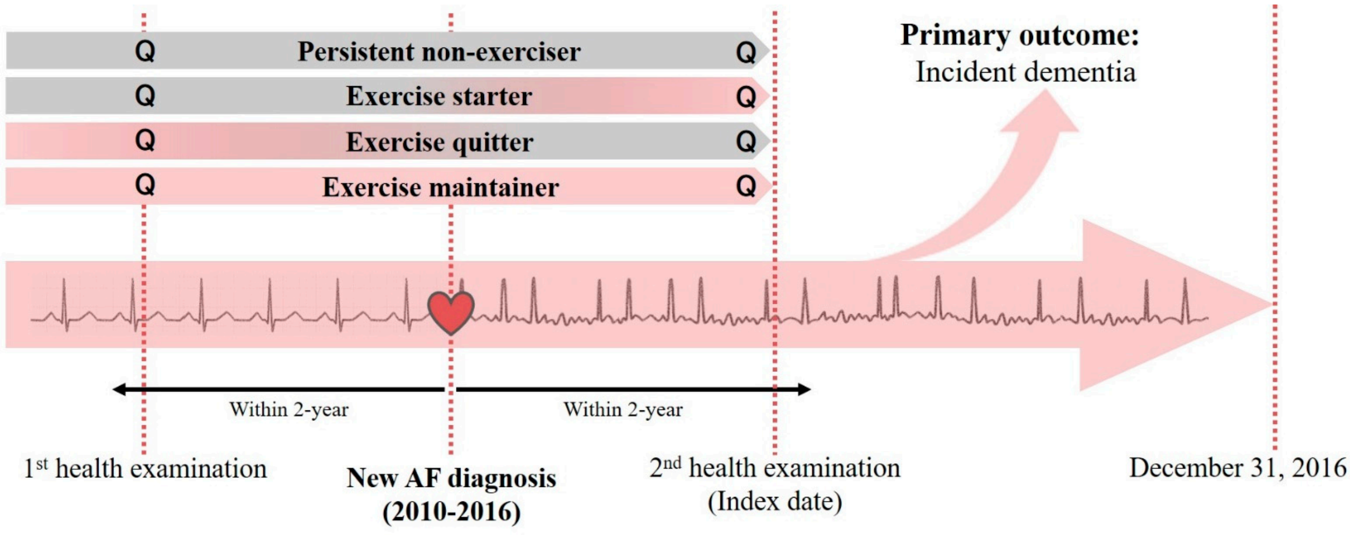

** Q = self-reported questionnaire from health examination to assess patient's level of physical activity.

Figure S2. Overall study flow.
